# Development and Administration of a Skin Survey Questionnaire for Methamphetamine Users

**DOI:** 10.7759/cureus.97266

**Published:** 2025-11-19

**Authors:** Brynne E Tynes, Kevin S Murnane, Mary F Vest, Fatih Gelir, M. Farris Sawaya, Mohammad Alfrad Nobel Bhuiyan, Sarah Murnane

**Affiliations:** 1 School of Medicine, Louisiana State University Health Sciences Center, Shreveport, USA; 2 Psychiatry and Behavioral Sciences, Louisiana State University Health Sciences Center, Shreveport, USA; 3 Pharmacology, Louisiana State University Health Sciences Center, Shreveport, USA; 4 Medicine, Louisiana State University Health Sciences Center, Shreveport, USA; 5 Internal Medicine, Louisiana State University Health Shreveport, Shreveport, USA; 6 Physical Therapy, Louisiana State University Health Sciences Center, Shreveport, USA

**Keywords:** formication, hyperpigmentation, inpatient, methamphetamine, picking, questionnaire, scarring, survey, wound

## Abstract

Cutaneous complications of methamphetamine use are frequently under-recognized in clinical settings. Increased detection could facilitate the early identification of dermatologic issues and support a more holistic recovery process. This study aimed to develop and implement a skin survey questionnaire to identify common dermatologic conditions among individuals with methamphetamine use disorder. A novel skin survey was administered to 94 participants enrolled in a residential treatment program. The questionnaire included sliding-scale and dichotomous response items addressing skin appearance, symptoms, and behaviors. Participants reported a range of skin issues, including track marks (n = 21; 22.34%), hyperpigmentation (n = 18; 19.15%), and scarring (n = 16; 17.02%). Average sliding-scale scores indicated delayed wound healing (X̄ = 0.307), minimal itchiness (X̄ = 0.229), and nominal skin picking (X̄ = 0.185). Notably, 13.83% (n = 13) reported formication. Higher average skin symptom scores were significantly associated with younger age (p = 0.0499), female sex (p = 0.0462), and non-smoking routes of methamphetamine administration, particularly IV injection (p < 0.001). Duration of methamphetamine use was not significantly correlated with skin score. The survey effectively identified a variety of dermatologic symptoms associated with methamphetamine use, supporting its utility as a clinical screening tool in residential treatment settings. Further validation of the tool is recommended for broader implementation.

## Introduction

Methamphetamine use is a public health crisis in the United States, with escalating rates, hospitalizations, and overdose deaths [1]. According to the National Survey on Drug Use and Health (NSDUH), 2.5 million people 12 years of age or older reported methamphetamine use in 2021 [2]. Clinicians across disciplines are familiar with the multiple health concerns that arise from substance use, such as infectious diseases like HIV and hepatitis C, driven in part by injection drug use, as well as high-risk behaviors [3] and neuropsychiatric consequences, including anxiety, depression, psychosis, and cognitive decline [4]. Less frequently discussed, but equally concerning, are dermatologic consequences of methamphetamine use that not only pose risks to physical health but may also exacerbate psychological distress and carry a social stigma, hindering recovery efforts.

Cutaneous manifestations of drug addiction, such as track marks, skin popping scars, infection, and pruritus, are common among methamphetamine users [5]. These visible indications of drug use may impact a person’s self-image, mental health, and social functioning, potentially negatively impacting recovery. Despite their prevalence, cutaneous manifestations of methamphetamine use are often overlooked in substance use treatment settings. For psychiatrists, dermatologists, and addiction specialists, recognizing and addressing these cutaneous manifestations presents an opportunity to improve patient outcomes. Developing a brief skin survey questionnaire for patients entering residential treatment could facilitate the identification and management of methamphetamine-related skin conditions and their emotional sequelae, as well as mitigate the physical reminders of addiction that may otherwise contribute to a negative self-perception. Early recognition and treatment of these cutaneous conditions may support improved recovery, prognosis, and psychological well-being.

Common cutaneous conditions in methamphetamine users

Scars and hyperpigmentation at injection sites, often referred to as “track marks,” are common skin manifestations of intravenous (IV) drug use. These lesions result from repeated injection, which causes thrombosis and damage to the veins, leading to scarring of the vessel and hyperpigmentation of the skin covering it [5]. Direct irritation from chronic injection, dull needles, or methamphetamine itself can all contribute to the development of track marks. Track marks typically appear on the nondominant arm at the antecubital fossa, but may also be found anywhere drugs are injected, such as the popliteal fossa, dorsal feet, or inguinal region.

Beyond IV use, individuals may also inject methamphetamine subcutaneously or intradermally, a practice known as "skin popping." This method often results in long-lasting tissue damage and hypopigmented, atrophic scars at injection sites [5]. Keloid formation, or raised scar tissue, may also develop. Original reference for photo examples of the aforementioned types of scarring is found in [5].

In addition to the cutaneous manifestations of the injection itself, methamphetamine is an immunosuppressant, impairing the function of neutrophils, macrophages, cytokines, and other immune mediators [6]. As a result, methamphetamine users are more susceptible to skin and soft tissue infections, including cellulitis, impetigo, necrotizing fasciitis, and abscesses. Cellulitis and abscesses are experienced by 22-65% of IV drug users, while 31% develop soft tissue sepsis [7]. Entry sites for cutaneous infections not only include injection sites, but also wounds created by picking [6]. Common pathogens include *Staphylococcus aureus*, methicillin-resistant *S. aureus *(MRSA), and streptococci [7]. In addition to its immunosuppressive effects, methamphetamine augments biofilm formation of *S. aureus*, enhancing the infection process [6]. Management may require treatment with antibiotics, drainage of pus, and even radiologic imaging for the identification of foreign bodies (e.g., needle fragments) [7].

Methamphetamine is also known to negatively affect wound healing. Chronic inflammation, repeated trauma, and the drug’s direct tissue-damaging effects hinder the cellular repair process [7]. Preclinical studies show that methamphetamine delays wound healing, with methamphetamine treated mice exhibiting prolonged inflammation and wound enlargement over time compared to untreated controls [6]. Persistent wounds and ulcers at injection sites are also common in long-term users [7].

Pruritus, or itching, is another frequently reported symptom in methamphetamine users [7]. Bang and colleagues (2023) found that 10% of methamphetamine users treated at San Francisco dermatology clinics from 2011 to 2021 received a pruritic diagnosis [8]. These included generalized (widespread) pruritis and prurigo nodularis, characterized by intensely pruritic, hyperkeratotic nodules commonly found on the trunk and extensor surfaces caused by excessive scratching or picking [9]. Patients affected by this inflammatory disease often experience an uncontrollable “itch-scratch cycle,” exacerbating the condition and causing bleeding or crusting.

Bang and colleagues (2023) also found that eczema was the most common cutaneous disorder among methamphetamine users, with a prevalence of 12% [8]. Characterized by inflamed, red, and itchy skin, eczema can worsen with scratching, leading to oozing, crusting, or scaling [10]. In adults, it commonly affects flexor surfaces, as well as the neck, hands, and feet.

Methamphetamine use is also linked to psychocutaneous disorders. Bang and colleagues (2023) reported that 9% of methamphetamine users were diagnosed with psychocutaneous disorders and were 21.8 times more likely to experience delusions of parasitosis compared to non-methamphetamine users [8]. For example, formication is a tactile hallucination of insects crawling on or underneath the skin that can result in compulsive scratching and clawing, creating open wounds or sores [5,7]. Successful treatment can often be achieved with drug cessation and antipsychotic medications [7].

Additional cutaneous effects of methamphetamine include hyperhidrosis (excessive sweating), xerosis (dry skin), premature skin aging, and strong body odor, all of which may further affect self-esteem and psychological well-being [5].

Psychological burden of skin conditions

Although research examining the psychological impact of dermatologic conditions in individuals with substance use disorders is limited, a growing body of evidence suggests a strong link between cutaneous disease and poor mental health in the general population. Visible skin conditions can invite social stigma, low self-esteem, and psychological distress-factors that may be further exacerbated in the context of addiction recovery. In a large European study by Dalgard et al. (2015) involving 3,635 patients with skin disease and 1,359 healthy controls, patients with dermatologic conditions were significantly more likely to experience clinical depression (10.1% vs. 4.3%), anxiety (17.2% vs. 11.1%), and suicidal ideation (12.7% vs. 8.3%) [11]. Over half (53.6%) of dermatology patients with suicidal thoughts reported that their skin condition was the direct cause. Among those with atopic dermatitis, 68% attributed suicidal ideation to their disease. Conditions most associated with depression included psoriasis, ulcers, eczema/atopic dermatitis, and skin infections - all commonly reported among methamphetamine users. Yu and Silverberg (2015) found that one in three adults with atopic dermatitis met diagnostic criteria for major depressive disorder [12]. Depression and anxiety were also found to have a significant positive correlation with a diagnosis of prurigo nodularis, another skin condition common among methamphetamine users [13].

An analysis by Pärna et al. (2015) investigated the broader psychosocial impact of chronic skin disease, including psoriasis, eczema, acne, and seborrheic dermatitis [14]. Compared to healthy controls, affected individuals experienced significantly higher levels of depression, general and social anxiety, panic-agoraphobia, fatigue, and insomnia. These findings suggest that skin disease may lead to social withdrawal, strained interpersonal relationships, impaired sleep, decreased self-confidence, and diminished quality of life. A total of 86% of patients in the study reported that their skin condition negatively affected their quality of life based on Dermatology Life Quality Index (DLQI) scores.

Individuals with substance use disorders who experience cutaneous complications may experience these psychological burdens, potentially exacerbating the already existing struggles of addiction. Not only are skin conditions an important physical manifestation of drug use and present psychological challenges on their own, but shame and stigma may surround the addiction and reduce the drive for abstinence. Individuals may believe that these visible signs are permanent, leading to decreased motivation to pursue treatment. Addressing dermatologic conditions in methamphetamine users may offer an opportunity to mitigate these psychological burdens. The current study aims to identify and characterize cutaneous manifestations of methamphetamine use with the ultimate goal of facilitating early recognition and treatment. We propose that a brief skin survey, administered upon admission to residential treatment, could efficiently identify distressing or clinically significant skin issues without requiring a full dermatologic examination. Such a tool could help clinicians allocate attention to relevant skin concerns, reduce the psychosocial impact of visible drug-related complications, and support a more comprehensive and holistic approach to addiction recovery.

## Materials and methods

Development of the survey

The survey was developed through a comprehensive review of existing validated instruments and current literature. The “Personal and Family History of Psoriasis” protocol from the “Skin” section of the PhenX Toolkit served as a structural guide [15]. To generate the list of substances for participant selection, the “Substances - Lifetime Abuse and Dependence” protocol from the “Alcohol, Tobacco, and Other Substances” section of the PhenX Toolkit was adapted [16].

For content development, a targeted PubMed search using the keywords “methamphetamine skin dermatology” was conducted to identify relevant literature. Key references included “Distribution of Skin Diseases Among Patients Using Methamphetamine” by Bang and colleagues (2023) [8], “The Effects of Alcohol and Illicit Drug Use on the Skin” by Jain and colleagues (2021) [7], and “Illicit Drugs: What Dermatologists Need to Know” by Hennings and Miller (2013) [5]. An additional PubMed search for “methamphetamine skin infection” identified “Methamphetamine Alters the Antimicrobial Efficacy of Phagocytic Cells During Methicillin-Resistant *Staphylococcus aureus* Skin Infection” by Mihu and colleagues (2015) [6], which informed the survey’s focus on skin infections and wound healing.

**Figure 1:**
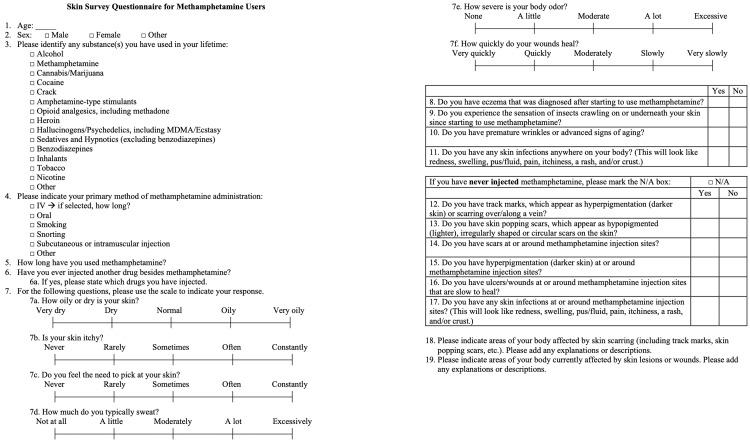
Skin survey administered to methamphetamine users in residential treatment This figure displays the questionnaire given to subjects in a residential treatment facility. The questionnaire consists of 19 items.

The survey was then reviewed for clinical sensitivity by multiple psychiatrists in the Louisiana State University Health Shreveport Department of Psychiatry to ensure that no items were judged likely to trigger distress among participants. Further expert reviews were conducted by a Louisiana State University dermatologist and a Certified Wound Specialist. The final skin survey questionnaire is presented in Figure 1.

Administering the survey

The survey was administered to residents of a local residential treatment facility in Shreveport, Louisiana. The inclusion criteria included English-speaking individuals admitted to a 30-day residential treatment program for methamphetamine use disorder who were able to read the informed consent document and complete the survey. Exclusion criteria included adults unable to provide informed consent, individuals under 18 years of age, and individuals temporarily released from incarceration for the purpose of receiving substance use treatment. Eligible participants were recruited following their admission to the program. Over a 10-month period, all newly admitted residents who met the inclusion criteria were invited to participate. Upon reviewing the approved consent letter, the survey was administered orally by trained research personnel. No identifying information was collected. Consented subjects were assigned a consecutive study number by the REDCap electronic data capture system, where responses were subsequently recorded and securely stored. This study was reviewed and approved by the Louisiana State University Health Sciences Center - Shreveport Institutional Review Board (approval no. STUDY00002432).

Data analysis

Descriptive statistics, including frequencies, sums, and percentages, were calculated using Microsoft Excel (Microsoft Corp., Redmond, WA, USA). An average skin score was calculated for each participant by aggregating responses from all skin-related survey questions. For items using a sliding scale format, REDCap automatically assigned values ranging from 0 to 1 based on the participant’s selected point on the scale. Dichotomous (Yes/No) responses were coded as 1 for “Yes” and 0 for “No”. Each participant's average skin score was calculated by taking the mean of all coded skin-related responses. These scores were then analyzed in relation to demographic and clinical variables, such as age, gender, and method of drug administration via regression and correlation analysis. Independent samples t-tests were used to compare mean skin scores between two groups, such as gender-based comparisons. For comparisons involving more than two independent groups, such as average skin scores across different routes of methamphetamine administration, a one-way analysis of variance (ANOVA) was conducted. When statistically significant differences were identified, Tukey’s Honest Significant Difference (HSD) post-hoc test was applied to perform pairwise comparisons and determine the specific group differences. All graphical data, including error bars and simple linear regression lines of best fit, were generated using GraphPad Prism (GraphPad Software, Inc., La Jolla, CA). Statistical significance was set at p < 0.05.

## Results

During the study period, 107 individuals met the inclusion criteria to complete the survey. Of these, 13 participants reported no history of methamphetamine use and were subsequently excluded from the analysis, resulting in a final sample size of 94 participants. Among these, 54 were identified as male (57.45%) and 40 as female (42.55%) (Table 1).

**Table 1:** Demographic characteristics of the participants (n = 94)

Demographic characteristic	n	%
Sex		
Male	54	57.45
Female	40	42.55
Race		
European American/White	71	75.53
African American	16	17.02
Native American	2	2.13
Hispanic	1	1.06
Other	4	4.26
Sexual orientation		
Heterosexual	85	90.43
Bisexual	5	5.32
Homosexual	4	4.26
Method of methamphetamine administration		
Smoking	49	52.13
Intravenous	30	31.91
Nasal insufflation	12	12.77
Oral	3	3.19

With respect to racial demographics, the majority of participants identified as European American/White (n = 71; 75.53%), followed by African American (n = 16; 17.02%), Hispanic (n = 1; 1.06%), and “Other” (n = 4; 4.26%). In terms of sexual orientation, most participants identified as heterosexual (n = 85; 90.43%), while five identified as bisexual (5.32%) and four as homosexual (4.26%).

Regarding the route of methamphetamine administration, smoking was the most commonly reported route (n = 49; 52.13%), followed by intravenous injection (n = 30; 31.91%), nasal insufflation (n = 12; 12.77%), and oral ingestion (n = 3; 3.19%). No participants reported subcutaneous or intramuscular injection.

**Figure 2:**
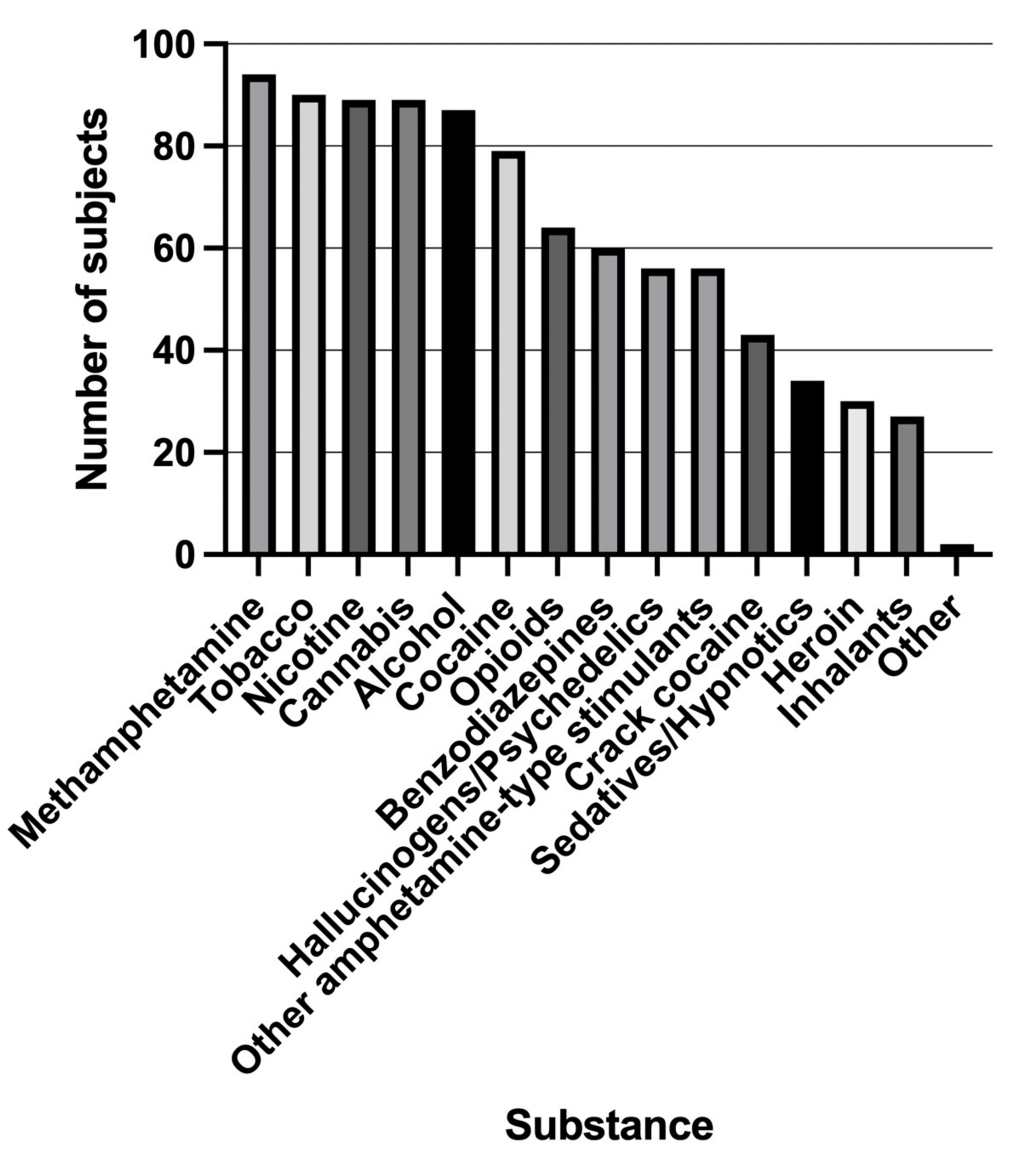
Distribution of lifetime substance use among the study participants (n = 94) Bar graph showing the frequency of participants using a substance at least once in their lifetime. The x-axis represents various substances, and the y-axis shows the number of participants who reported using it. All 94 subjects reported use of methamphetamine.

The participants were presented with a comprehensive list of substances and asked to indicate which they had used at least once in their lifetime (Figure 2). All 94 participants reported use of methamphetamine (100%). In addition, 90 participants (95.74%) reported tobacco use, 89 (94.68%) reported use of cannabis/marijuana or nicotine products, and 87 (92.55%) reported alcohol use. Cocaine use was reported by 79 participants (84.04%), and 64 (68.09%) indicated use of opioid analgesics.

Benzodiazepine use was reported by 60 participants (63.83%), while 56 participants (59.57%) endorsed use of amphetamine-type stimulants or hallucinogens/psychedelics, and the same number (n = 56; 59.57%) reported using sedatives/hypnotics. Crack cocaine use was reported by 43 individuals (45.74%), inhalant use by 34 (36.17%), and heroin use by 30 (31.91%). Two participants (2.13%) reported the use of other substances not included in the main categories.

Notably, nine participants (9.57%) reported having used every substance listed in the survey at some point in their lives. In addition, 26 participants (27.66%) reported injecting one or more substances other than methamphetamine in their lifetime.

**Figure 3:**
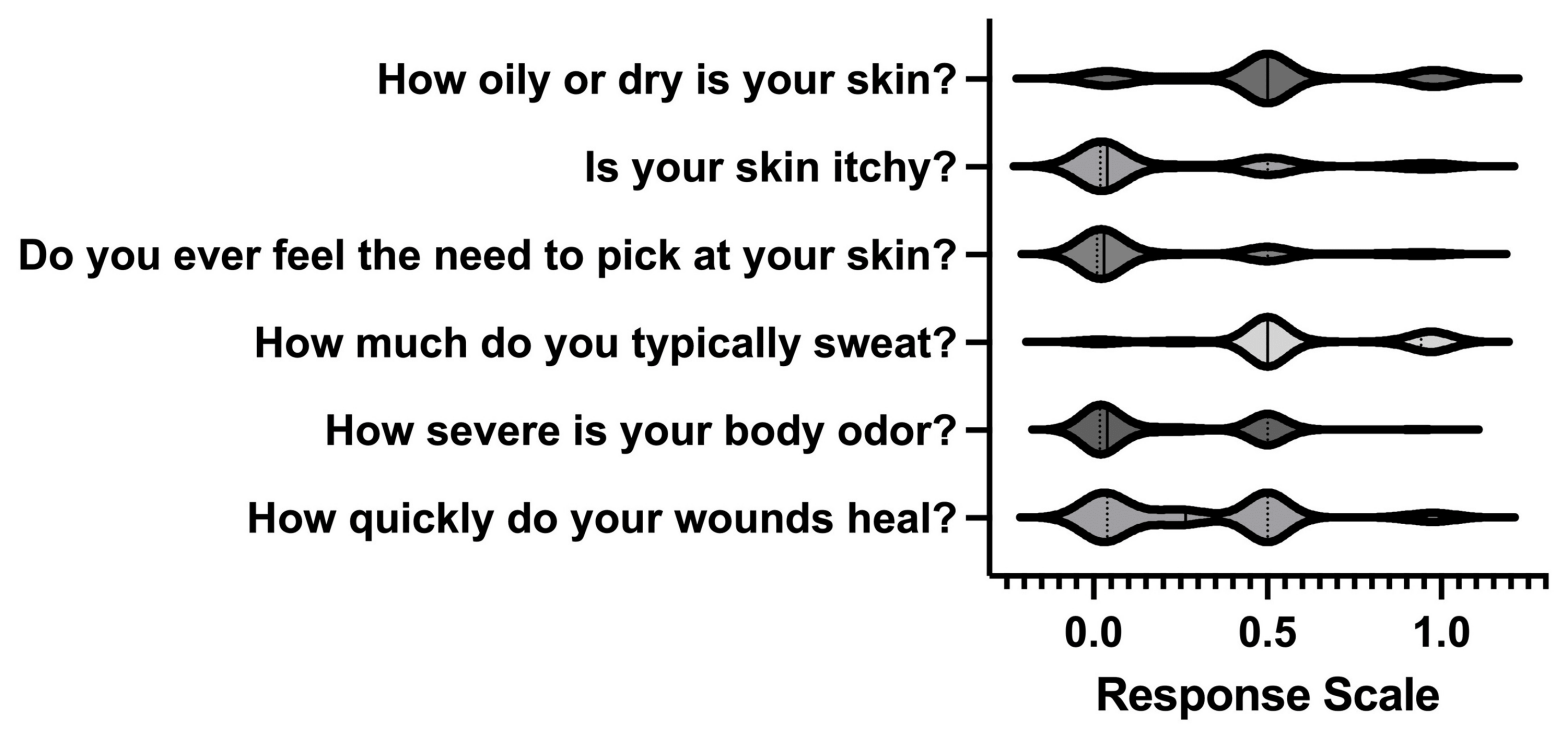
Violin graph of response density to the perception-based sliding scale items (n = 94) The width of each violin corresponds to the data density, with wider sections indicating higher data frequency. The solid lines represent the median and the dotted lines represent the interquartile range.

Several items on the survey utilized sliding scale response formats (Figure 3). For each scale, values ranged from 0 to 1, with 0 indicating the least severe or absent condition and 1 indicating the most severe or frequent condition. The mean response for self-perceived skin oiliness or dryness (0 = very dry, 1 = very oily) was 0.505, suggesting a neutral average. For skin itchiness (0 = never itchy, 1 = constantly itchy), the average score was 0.229. When asked about the compulsion to pick at their skin (0 = never, 1 = constantly), the mean response was 0.185.

Regarding perspiration levels (0 = not at all, 1 = excessively), participants reported a mean score of 0.594, indicating a tendency toward moderate to excessive sweating. The average response for the severity of body odor (0 = none, 1 = excessive) was 0.210. Lastly, participants rated their wound healing speed (0 = very quickly, 1 = very slowly) with an average score of 0.307.

Responses to dichotomous (Yes/No) questions are summarized in Table 2. Additional skin concerns reported in the free-text response section included abscesses, boils, and open sores.

**Table 2:** Frequency of “Yes” responses to binary survey questions (n = 94), with n representing the number of subjects answering “Yes”

Yes/No question	n	%
Do you have eczema that was diagnosed after starting to use methamphetamine?	4	4.26%
Do you experience the sensation of insects crawling on or underneath your skin since starting to use methamphetamine?	13	13.83%
Do you have premature wrinkles or advanced signs of aging?	15	15.96%
Do you have any skin infections anywhere on your body?	3	3.19%
Do you have track marks, which appear as hyperpigmentation (darker skin) or scarring over/along a vein?	21	22.34%
Do you have any skin popping scars, which appear as hypo- pigmented (lighter), irregularly shaped or circular scars on the skin?	1	1.06%
Do you have scars at or around methamphetamine injection sites?	16	17.02%
Do you have hyperpigmentation (darker skin) at or around methamphetamine injection sites?	18	19.15%
Do you have ulcers/wounds at or around methamphetamine injection sites that are slow to heal?	3	3.19%
Do you have any skin infections at or around methamphetamine injection sites?	2	2.13%

**Figure 4:**
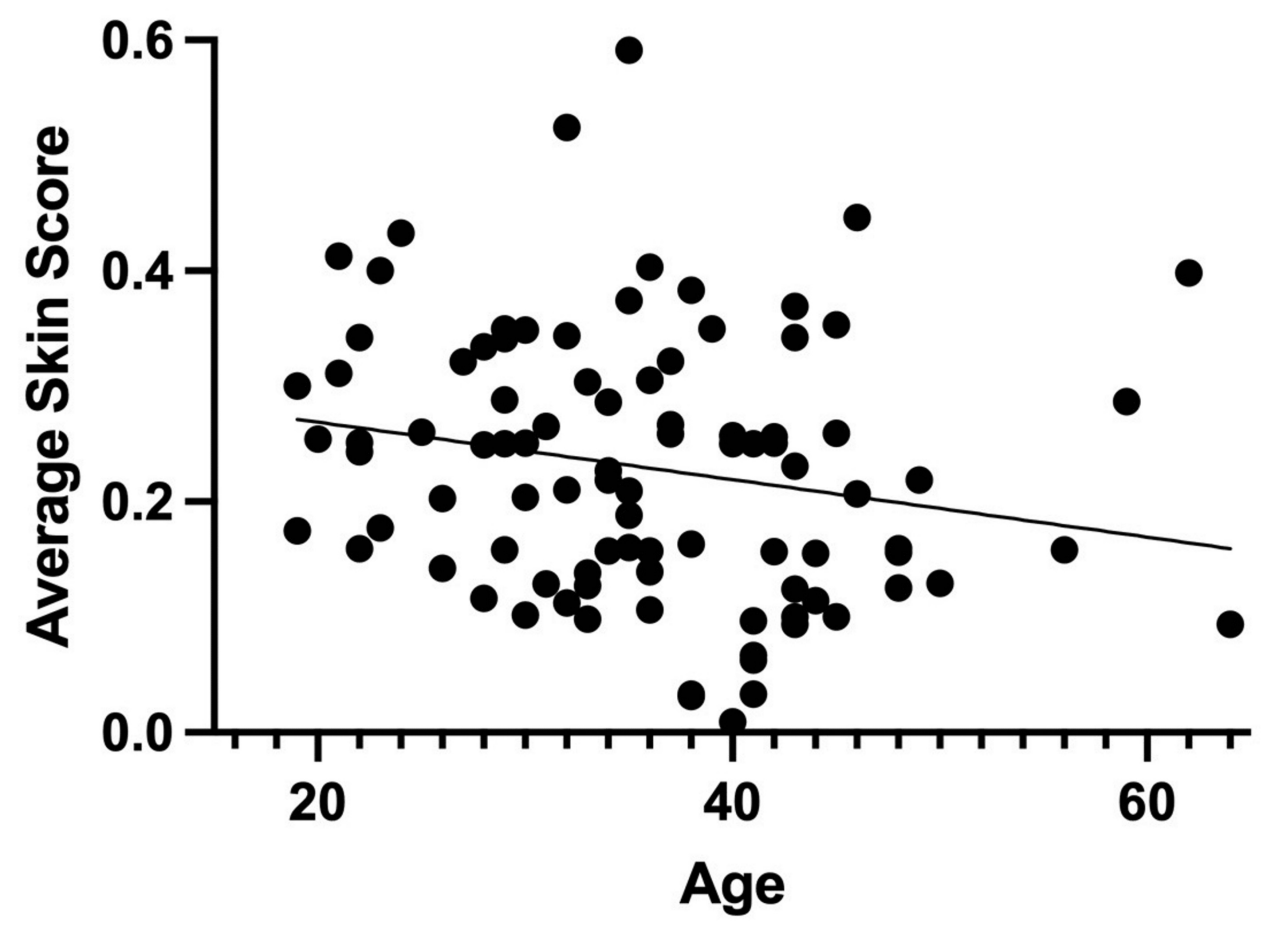
Correlation between participant age and average skin score with a linear trendline (n = 94) Scatter plot illustrating the relationship between the age of participants and their corresponding average skin condition score. The x-axis represents participant age, and the y-axis represents the average skin condition score as defined in the methodology section. A linear trendline is included to visualize the overall direction and strength of the correlation between age and skin condition score, indicating a potential decrease in score with increasing age. Individual data points represent each participant's age and score. Statistical significance was determined using Pearson correlation analysis (p < 0.05).

**Figure 5:**
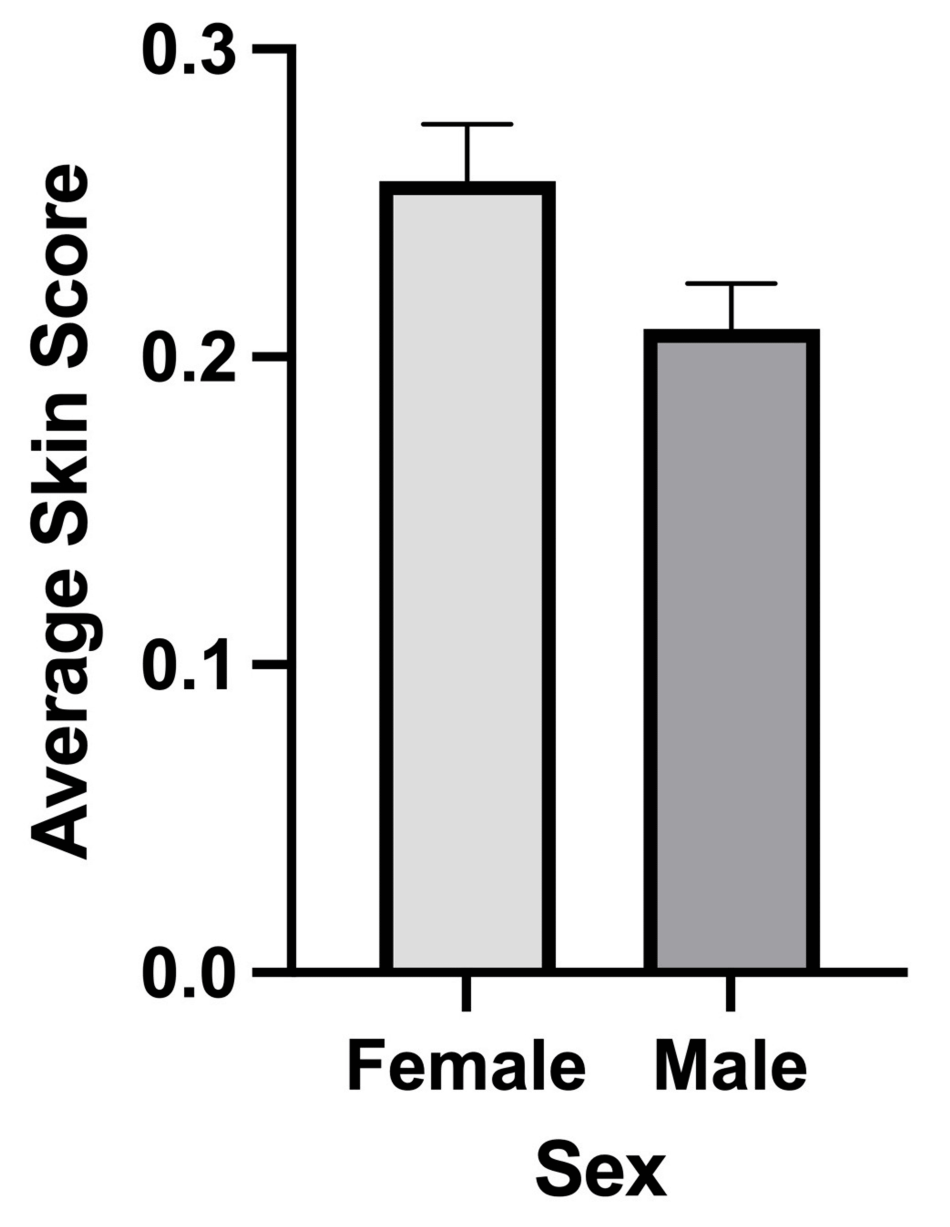
Comparison of average skin scores between sexes (n = 94) Bar graph showing the mean average skin scores for male and female participants. Error bars denote the standard error of the mean. Independent t-test revealed a significant difference between sexes, with p = 0.0462.

Several significant associations were identified between average skin scores and participant characteristics. Age demonstrated a statistically significant inverse relationship with average skin score, with older participants reporting lower scores (p = 0.0499; Figure 4). By contrast, duration of methamphetamine use was not significantly correlated with average skin score (p = 0.311). Sex was also significantly associated with the average skin score. Female participants exhibited higher average skin scores (X̄ = 0.257) compared to male participants (X̄ = 0.209), a difference that reached statistical significance (p = 0.0462; Figure 5).

**Figure 6:**
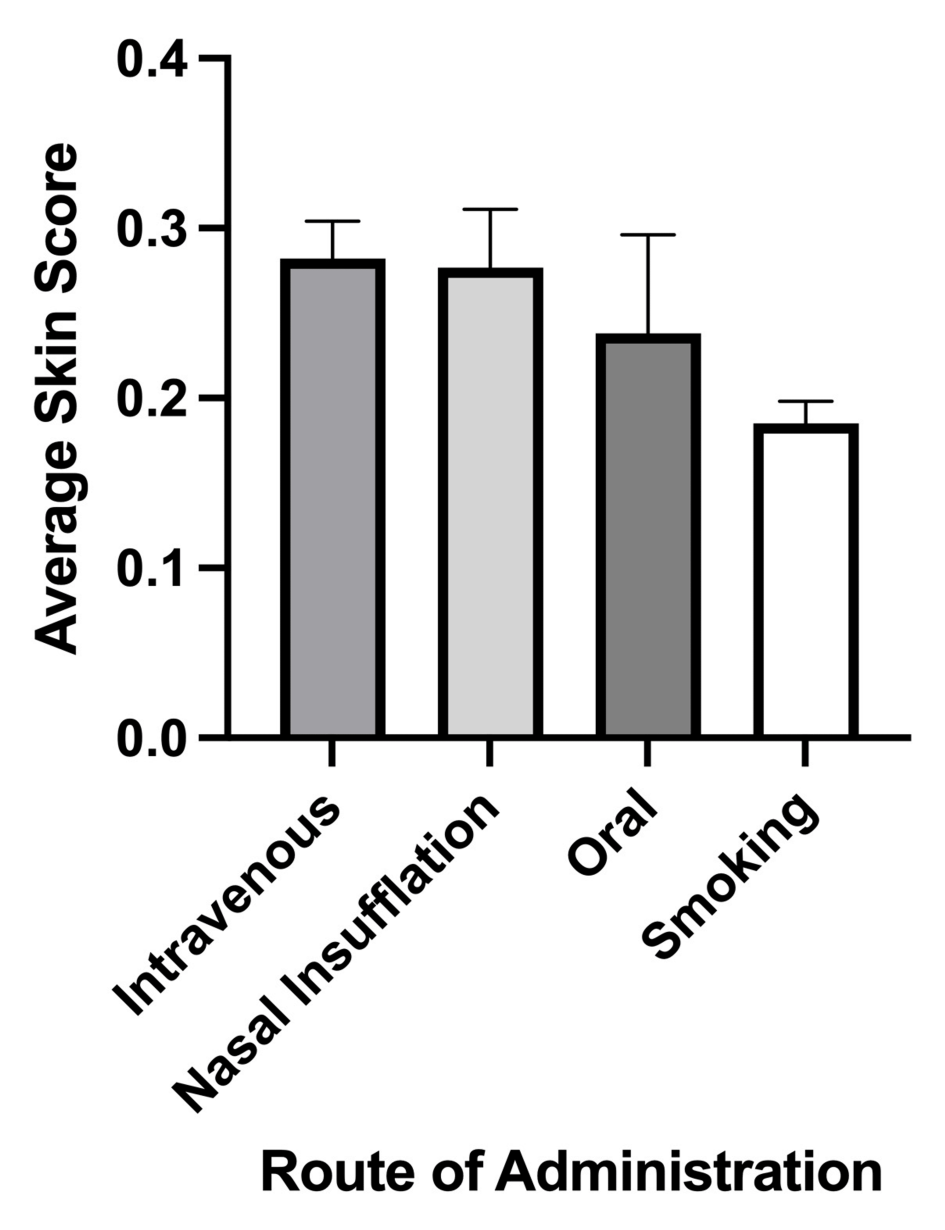
Average skin scores by primary methamphetamine administration method (n = 94) Bar graph comparing the average skin score across different modes of methamphetamine administration. Error bars denote the standard error of the mean. Statistical significance was determined using a one-way analysis of variance (ANOVA) (p < 0.05).

A significant relationship was found between the average skin score and primary method of methamphetamine administration (p = 0.001). Tukey’s HSD post-hoc comparison revealed that individuals who primarily used methamphetamine via IV injection (X̄ = 0.282, p < 0.001) or insufflation (X̄ = 0.277, p = 0.0415) had significantly higher skin scores than those who primarily smoked the drug (X̄ = 0.185). Participants who reported oral use had an average skin score of 0.238 (Figure 6). No significant associations were found between average skin score and either race (p = 0.343) or sexual orientation (p = 0.133).

## Discussion

In line with its potential effects on the skin, methamphetamine affects organ systems beyond the brain [17-19]. The drug is known to produce profound oxidative stress and inflammation that could be a key mechanism in its adverse dermatological effects [17,18,20,21]. In addition, methamphetamine is often taken by individuals from socially vulnerable communities [22], and it is known that individuals from such communities are also susceptible to dermatological disease through both social and biological disparities [23]. The findings of this study support the initial hypothesis that the development and implementation of a skin survey questionnaire in a residential treatment setting can effectively identify cutaneous conditions associated with methamphetamine use. Many of the conditions identified, such as psychocutaneous disorders, various skin infections, and other dermatologic manifestations, are both common and treatable. These results underscore the clinical utility of incorporating a standardized screening tool to facilitate early detection and intervention for skin-related concerns in this population.

Demographics

Although the skin survey was administered at a single residential treatment facility, the sample reflected a diverse demographic profile. Both sexes were adequately represented, with 57.45% male (n = 54) and 42.55% (n = 40) female participants. While the majority identified as heterosexual (n = 85; 90.43%), sexual minorities, including bisexual (n = 5; 5.32%) and homosexual (n = 4; 4.26%) individuals, were also represented. Racial composition was predominantly European American/White (n = 71; 75.53%), with African American individuals comprising 17.02% (n = 16), and smaller percentages identifying as Hispanic (n = 1; 1.06%) or “other” (n = 4; 4.26%).

Participants reported a variety of methamphetamine administration routes, with smoking being most common (n = 49; 52.13%), followed by IV injection (n = 30; 31.91%), nasal insufflation (n = 12; 12.77%), and oral use (n = 3; 3.19%). This distribution allowed for meaningful comparisons across different administration methods.

Survey responses

On average, participants reported neutral skin characteristics--neither particularly oily nor dry (X̄ = 0.505), mild body odor (X̄ = 0.210), and moderate sweating (X̄ = 0.594). These findings suggest that widespread symptoms such as hyperhidrosis, xerosis, or severe body odor, often reported in the literature, may not be as prevalent among this cohort or less likely in non-intoxicated patients [5]. Similarly, the average scores for itchiness (X̄ = 0.229) and compulsive skin picking (X̄ = 0.185) were relatively low, indicating that most participants did not frequently experience these issues. While these results were not consistent with higher rates of pruritus seen in the literature [7,8], a subset of participants did report increased sweating and urges to scratch or pick at their skin (Figure 4), highlighting the importance of retaining these items to capture at-risk individuals. Additionally, the mean score for perceived wound healing speed (X̄ = 0.307) indicated that many participants felt their wounds healed slowly, which is consistent with prior research demonstrating methamphetamine’s detrimental effects on wound healing [7].

The most frequently reported dermatologic concern was track marks (n = 21; 22.34%), followed by hyperpigmentation (n = 18; 19.15%) and scarring at injection sites (n = 16; 17.02%). These findings point to the visibility of substance use through dermatologic sequelae. This may affect patients' self-image post-treatment, especially when co-existing with other noticeable non-cutaneous effects of methamphetamine, such as those on dentition, including excessive caries, decay, and missing teeth [24]. Premature aging (n = 15; 15.96%) was also reported, potentially compounding these psychosocial challenges. Furthermore, the negative effects of methamphetamine use on biological aging have been shown to significantly decrease facial symmetry compared to controls, even impairing the effectiveness of facial recognition technology [25].

Notably, 13.83% (n = 13) of participants reported experiencing formication since initiating methamphetamine use. This is slightly higher than the 9% prevalence reported by Bang and colleagues [8]. Given its association with psychocutaneous disorders, which can lead to excoriation, scarring, and secondary infections, identifying this symptom is essential for timely psychiatric and dermatologic intervention [5,7]. The ability of the skin survey to detect such symptoms highlights its potential utility in clinical screening protocols.

Other dermatologic manifestations, such as eczema, ulcers, and infections, were less frequently reported in this sample compared to previous studies. For instance, only 4.26% (n = 4) of participants endorsed a new eczema diagnosis following methamphetamine use, compared to 12% reported by Bang and colleagues [8]. However, several participants reported issues such as abscesses, boils, and open sores in free-response items, underscoring the need for early identification and treatment of potentially serious infections, even when prevalence is low.

Correlations and relationships

As hypothesized, individuals who primarily used methamphetamine intravenously exhibited the highest average skin scores, indicating more frequent or severe dermatologic concerns. Participants who reported insufflation also had significantly elevated skin scores compared to those who primarily smoked, suggesting that the route of administration plays a role in cutaneous manifestation and should be considered during assessment.

Interestingly, female participants had significantly higher average skin scores than males. While this may reflect an actual increased incidence in skin condition prevalence, it could also suggest increased awareness, concern, or willingness to report such symptoms among women. Similarly, younger individuals reported significantly higher average skin scores, which may be due to greater engagement in high-risk behaviors or differences in perception and self-reporting. Notably, duration of methamphetamine use was not significantly correlated with skin scores, suggesting that factors other than chronicity may play a more prominent role in the reporting of dermatologic outcomes.

Limitations

This study has several limitations. First, the data rely solely on self-reported survey responses, without corroboration from a clinical examination. Consequently, the presence or severity of skin conditions could not be objectively verified. In addition, the absence of a control group limits the ability to attribute skin concerns specifically to methamphetamine use, as participants frequently endorsed use of other substances such as tobacco, cannabis, cocaine, and opioids. Approximately one-quarter of the sample reported injecting substances other than methamphetamine, further complicating attribution of dermatologic symptoms to methamphetamine use alone.

The sample size (n = 94) was modest and recruited from a single treatment center, limiting generalizability. Furthermore, the survey instrument used in this study was newly developed and has not yet undergone formal validation. These factors suggest caution in interpreting the findings and highlight the need for replication and validation in larger, more diverse populations.

Future directions

Further research is warranted to validate the skin survey instrument and assess its reliability and clinical utility in identifying dermatologic concerns among individuals with methamphetamine use disorder. Larger studies across multiple treatment centers, with broader demographic representation, are needed to enhance generalizability and reduce potential confounding variables. The inclusion of a control group would help delineate the specificity of cutaneous manifestations linked to methamphetamine use.

Incorporating objective measures such as dermatologic examinations or photographic documentation would strengthen future studies and help validate self-reported data. Additionally, assessing the psychosocial impact of skin conditions-such as stigma, self-esteem, and employment outcomes-could provide important insights into the broader consequences of cutaneous complications in substance use recovery. Methamphetamine lacks either pharmacotherapeutic [26] or interventional [27] treatments, suggesting that monitoring of dermatological changes may also be useful as a biomarker for therapeutic development. Ultimately, interventions aimed at addressing these dermatologic concerns may improve overall treatment outcomes and quality of life for individuals recovering from methamphetamine use disorder.

## Conclusions

Just as the brain and body require healing in recovery, so too does the skin; addressing visible signs of addiction may not only alleviate physical discomfort but also help restore dignity, reduce stigma, and support long-term psychosocial recovery. The use of a skin survey questionnaire upon intake to residential treatment facilities offers a promising approach for identifying these often-overlooked conditions, enabling timely intervention during treatment. Our findings support the utility of this tool, as it effectively identified common and treatable dermatologic issues in the study population.

While individuals who inject or insufflate methamphetamine, females, and younger participants were more likely to report skin concerns, universal administration of the survey is recommended to maximize detection across the population. As a screening tool, the survey provides a time-efficient method for highlighting concerns that may otherwise go unaddressed without requiring a full dermatologic examination for each patient. Further research is needed to evaluate the tool’s reliability, validity, and impact on treatment outcomes. Incorporating a validated skin survey into standard intake procedures may ultimately enhance clinical care, reduce complications, and improve the overall recovery experience for individuals with methamphetamine use disorder.
